# Studies on Microbial Quality, Protein Yield, and Antioxidant Properties of Some Frozen Edible Insects

**DOI:** 10.1155/2021/5580976

**Published:** 2021-03-23

**Authors:** Peter Kurdi, Patspon Chaowiwat, Jirathit Weston, Chanida Hansawasdi

**Affiliations:** Food Science and Technology, Science Division, International College, Mahidol University, Buddhamonthon 4 Rd, Nakhonpathom, Salaya 73170, Thailand

## Abstract

This research investigated the microbial quality and the protein contents of Thai commercial frozen products of silkworm (*Bombyx mori*), bamboo caterpillar (*Omphisa fuscidentalis*), and field cricket (*Gryllus bimaculatus*). Total mesophilic aerobic counts were about 8-8.4 log cfu/g, while lactic acid bacteria numbers were up to 5.2 log cfu/g samples. Yeasts and mold as well as *Enterobacteriaceae* numbers were found to be no more than 6.3 and 6.6 log cfu/g, respectively, while *Clostridium* spp. enumeration detected 3.2-3.6 cfu per gram frozen insect samples. The protein content in cases of cricket and bamboo caterpillar after the Soxhlet defatting method and the Folch lipid extraction combined with isoelectric point precipitation were similar when compared at pH 3.5 or pH 4.5. In contrast, the protein yield from silkworm was higher at pH 3.5 in the Soxhlet defatted sample, as opposed to the Folch method treated sample where higher protein yield was obtained at pH 4.5. Furthermore, 2,2-diphenyl-1-picryl-hydrazyl (DPPH) radical scavenging activity and ferric reducing antioxidant power (FRAP) of field cricket subcritical water (121 or 135°C for 15 or 30 minutes) hydrolysates were also measured on a pilot basis. These tests revealed higher antioxidant activities in treated samples than in the untreated control. The highest DPPH radical scavenging activity and FRAP values were detected in samples treated at 135°C, while the protein content of these samples was lower than that of the untreated control. These results indicate that hydrolytic compounds of proteins and probably other types of cricket materials are possibly involved in the antioxidant activities of the treated defatted cricket samples.

## 1. Introduction

It is widely accepted that the human population on planet Earth would reach 9 billion by 2050, and one of the huge challenges of the 21^st^ century is to produce enough food for everyone. For this reason, global interest towards alternative and novel food sources has been increased including the recent “rediscovery” of edible insects, especially in western societies. Growing insects has several economic and ecological advantages over traditional farm animals, such as less water, land, and feed requirements for their life cycles, high feed conversion efficiency, and rapid reproduction, as well as less greenhouse gas emission during the rearing process [1]. In many countries of Africa and Asia insects are traditional ingredients of the local cuisine being a good source of high-quality proteins, essential amino acids, and insoluble dietary fiber (chitin) as well as polyunsaturated fatty acids, minerals (e.g., Fe, Zn, and K), and vitamin B1 and B2 [1].

In Thailand, several kinds of edible insects are consumed as deep-fried snacks including bamboo caterpillar (*Omphisa fuscidentalis*), silkworm (*Bombyx mori*), and field cricket (*Gryllus bimaculatus*), which are also available at supermarkets in uncooked, frozen packages. Although these frozen products are generally perceived by Thai customers as safe, studies assessing the microbial load of these are lacking. This study is aimed to investigate the microbial quality of commercially available frozen bamboo caterpillar (*Omphisa fuscidentalis*), silkworm (*Bombyx mori*), and field cricket (*Gryllus bimaculatus*) products. Besides, protein extraction procedures after two different kinds of lipid removal methods were also investigated, potentially aiding technological processes aiming to extract and purify insect proteins from the above species as the most important nutritional component of edible insects.

Recently, subcritical water extraction is gaining increased attention as it utilizes water molecules as solvent and catalyst above both boiling temperature and atmospheric pressure. It is also a cost-effective and environmentally safe method that offers great potential to extract valuable, especially nonpolar compounds from biological samples. Additionally, novel nutritional benefits of edible insect were revealed by a recent work that reported antioxidant properties in both the liposoluble and water-extracts of insects [2]. Similarly, defatted flours and hydrolysates of several edible insects were also found to possess antioxidant activities by Botella-Martinez et al. [3] and Zielińska et al. [4], respectively. Therefore, as a novel approach, subcritical water extraction was applied on defatted field crickets to gain more insights about the antioxidant properties such as DPPH free radical scavenging activities and ferric reducing antioxidant power of these edible insects.

## 2. Materials and Methods

### 2.1. Materials

Frozen insects: silkworm (*Bombyx mori*), bamboo caterpillar (*Omphisa fuscidentalis*), and field cricket (*Gryllus bimaculatus*) were purchased from a local supermarket in Bangkok, Thailand. The purchased insect samples were stored at -4°C. Folin and Ciocalteu's phenol reagent, 2,2-diphenyl-1-picrylhydrazyl (DPPH), 2,4,6-Tris(2-pyridyl)-s-triazine (TPTZ), and bovine serum albumin (BSA) were purchased from Sigma-Aldrich (St. Louis, MO, USA). Iron (II) sulfate heptahydrate was purchased from Merck Millipore (Darmstadt, Germany). All the reagents used were of analytical grade.

### 2.2. Microbiological Analysis

Microbial analysis of frozen insect samples was based on the work of Fasolato et al. [5] and focused on total microbial count, lactic acid bacteria, yeasts and molds, *Enterobacteriaceae*, and *Clostridium perfringens*. Five grams of sample was weighed and mixed with 5 mL 0.9% saline solution. The mixture was then homogenized using a homogenizer (IKA T10 basic, IKA-Werke GmbH & Co., Staufen, Germany) equipped with sterilized blades for 15 minutes or until fully homogenized.

Aerobic mesophilic microorganisms were enumerated on plate count agar (PCA, HiMedia Laboratories, Mumbai, India) incubated at 30°C for 48 hours. *Enterobacteriaceae* were counted on violet red bile glucose agar (VRBG, HiMedia Laboratories), after incubation at 37°C for 24 hours. Lactic acid bacteria were enumerated on de Man, Rogosa, and Sharpe agar (MRS, Laboratorios Conda S.A., Madrid, Spain), incubated at 30°C for 48 hours. Yeasts and molds were counted on oxytetracycline glucose yeast extract (OGY) agar after incubation at 25°C for 5 days. OGY agar was prepared by mixing D-glucose (20 g/L), yeast extract (5 g/L), and bacteriological agar (15 g/L) with distilled water. The solution was autoclaved at 121°C for 15 minutes. Oxytetra Selective Supplement (HiMedia Laboratories) was then aseptically added to the autoclaved and cooled medium (45-50°C) before pouring plates. *Clostridium perfringens* was enumerated on tryptose sulphite neomycin agar (TSN, HiMedia Laboratories) at 46°C for 24 hours in anaerobic atmosphere (generated with Anaero Pack, Mitsubishi Gas Chemical Co., Tokyo, Japan). All determinations were conducted in triplicates.

### 2.3. Analysis of Crude Protein Content

Total protein content was determined by the Kjeldahl method. The method was performed according to method 981.10 of the AOAC [6]. All the insect samples were analyzed twice using a steam distillation system (Vapodest 20, C. Gerhardt GmbH & Co. KG, Königswinter, Germany). The amount of obtained total nitrogen of the insect samples was multiplied by species-specific conversion factor of 4.76 as recommended by Janssen et al. [7] in order to not overestimate the total protein content.

### 2.4. Lipid Extraction

#### 2.4.1. Soxhlet Extraction

Hot air-dried samples (5 g) were ground by blender before defatting using a Soxhlet apparatus (Biosolve, Dieuze, France) with 400 mL petroleum ether as a solvent following the official AOAC method (991.36; [6]). The extraction cycle ran for 3 hours for silkworm and for 2 hours 30 minutes for bamboo caterpillar and field cricket. The extracts were dried for 24 hours at room temperature to remove residual solvent. The defatted residues were kept in a desiccator before use.

#### 2.4.2. Folch Extraction

This lipid extraction method was adopted from Tzompa-Sosa et al. [8] based on the principle of phase separation between polar and nonpolar solvents. Ten grams of ground dried samples was prepared in each centrifuge bottle; then, 200 mL dichloromethane and methanol were added at a ratio of 2 : 1. The mixtures were shaken for 20 seconds, then sonicated in a benchtop sonicator washer for 10 minutes, and placed in a laboratory shaker water bath for 2 hours at 150 rpm in room temperature. Afterwards, 25 mL distilled water was added into the mixtures and centrifuged for 20 minutes at 10,000 x *g* in room temperature. After the centrifugation, there were 3 layers separated. The top layer, composed of nonlipid and polar components, was pipetted out by glass pipette. The middle layer, composed of lipid components dissolved in the solvent, was discarded. The bottom part composed of insoluble residues was also collected. The collected parts were cooled and dried for 24 hours at room temperature to remove residual solvent then later used as defatted protein in further protein isolations.

### 2.5. Protein Isolation Using Isoelectric Point Precipitation

The protein extraction method was a modified version of what was described by Gresiana et al. [9]. Eight grams of defatted insect powder was suspended in 80 mL of distilled water and stirred for 1 hour. The mixture was subjected to vacuum filtration, and the supernatant was later used in the protein isolation process. The pH of the crude protein extract was adjusted to 3.5 or 4.5 then stirred for 10 minutes followed by centrifugation for 10 minutes (10,000 x *g*, 4°C). (These pHs were proven to yield the most protein amounts among the pHs tested in a preliminary experiment.) The pellets were collected and dried by hot air at 55-60°C for 1 hour. The dried protein isolate was weighed to calculate the yield percentage of isolation. The isolated protein was then grounded and dissolved in 2 M NaOH and distilled water in the ratio of 1 : 9, resulting in a total concentration of 1 mg/mL which was later used in Lowry protein assay (see below).

### 2.6. Protein Content Determination

The total protein content was measured by the Lowry method according to Waterborg [10]. The protein reagent was prepared by mixing 2% CuSO_4_.5H_2_O, 3% KNaC_4_H_4_O_6_.4H_2_O, and 3% Na_2_CO_3_ in 0.1 M NaOH (1 : 1). This solution was mixed in a 1 : 1 : 98 ratio prepared freshly before use. The insect protein isolate samples (0.2 mL) were mixed well with 2.0 mL of protein reagent and incubated at room temperature for 10 minutes. Afterwards, 0.2 mL of 50% Folin and Ciocalteu's phenol reagent was added into the mixture and incubated at room temperature for another 30 minutes. The absorbance of the samples was measured in a spectrophotometer (Spectronic 200, Thermo Scientific, Waltham, MA, USA) at 660 nm, and the protein concentration was determined using a standard curve made by measuring absorbances of bovine serum albumin solutions between 0 and 1 mg/mL concentrations. The results were analyzed by T-test at 95% confidence and presented as mean ± standard deviation (SD).

### 2.7. Hydrothermal Treatment of Defatted Crickets

Frozen whole field crickets (2 g) were defatted by the Soxhlet method as described above except that the extraction cycle was run for 1 hour. Defatted crickets were mixed in flasks with water by the ratio of 1 : 1. The flasks were then autoclaved in the following conditions: 121°C for 15 minutes, 121°C for 30 minutes, 135°C for 15 minutes, and 135°C for 30 minutes. All samples were cooled down and vacuum filtered to obtain supernatant of cricket hydrolysates and were subsequently freeze-dried. The protein content of the freeze-dried cricket hydrolysates was measured by the Lowry method as described above.

### 2.8. 2,2-Diphenyl-1-Picryl-Hydrazyl (DPPH) Radical Scavenging Assay

The DPPH free radical-scavenging activities of cricket hydrolysates were determined according to Bougatef et al. [11]. Freeze-dried cricket hydrolysate was dissolved in deionized water to have 1 mg/mL protein content, and this mixture was served as sample in the assay. 125 *μ*L of 0.02% DPPH in 99.5% ethanol was mixed with 1 mL of samples then subsequently incubated in the dark at room temperature for 60 minutes. Afterwards, the absorbance of the samples at 517 nm was measured by a spectrophotometer (Spectronic 200). A control assay was performed by adding distilled water instead of cricket hydrolysate. DPPH radical scavenging activity was calculated by the following equation: DPPH radical scavenging% = [(*A*_0_ − *A*_1_)/*A*_0_] x 100, where *A*_0_ is the absorbance of the DPPH solution (control) and *A*_1_ is the absorbance of the sample.

### 2.9. Ferric Reducing Antioxidant Power (FRAP) Assay

The FRAP assay of freeze-dried cricket hydrolysates was carried out according to the method of Rabeta and Faraniza [12]. The FRAP reagent was obtained by mixing 0.3 M acetate buffer (pH 3.6), 10 mM 2,4,6-Tris(2-pyridyl)-s-triazine (TPTZ) dissolved in 40 mM HCl, and 2 mM FeCl_3_ in a ratio of 10 : 1 : 1. Two hundred microliters of freeze-dried cricket hydrolysates dissolved in deionized water with 1 mg/mL protein content was mixed with 3 mL of FRAP reagent then incubated for 30 minutes in a water bath at 37°C. Afterwards, the absorbances of the sample mixtures were measured at 593 nm in a spectrophotometer (Spectronic 200). A standard curve was obtained by conducting the assay with different concentrations of FeSO_4_·7H_2_0, and the assay results were expressed as Fe^2+^ equivalents (mM) per gram of samples.

### 2.10. Statistical Analyses

Results are expressed as means ± standard deviations of triplicate analyses for each sample. Differences between samples that underwent different treatments were analyzed by applying Student's *t*-test. Data on protein content was statistically analyzed by One-way Anova followed by Duncan's multiple range test using SPSS Statistics (version 18). The level of significant difference was considered at a *p* value less than 0.05.

## 3. Results and Discussion

### 3.1. Microbiological Analysis of Frozen Bamboo Caterpillar, Field Cricket, and Silkworm

The results of microbial analyses of three Thai commercial frozen edible insect products are shown in Table 1. Total bacterial numbers in the three frozen samples were about 8-8.4 log cfu/g while lactic acid bacteria, yeasts and molds, *Enterobacteriaceae*, and *Clostridium* spp. numbers were in the range of 0-5.2, 5.6-6.3, 4.7-6.6, and 3.2-3.6 cfu/g, respectively. Total aerobic mesophilic bacterial numbers were found comparable to published data on fresh silkworm [5].

Prior quantitative microbial analyses on field cricket and bamboo caterpillar are lacking; however, comparing our data to published findings on other edible insects (reviewed by Garofalo et al. [13]), it can be concluded that the three frozen insect samples had moderate microbial load in the specific microbe groups that were investigated (total aerobic mesophiles, lactic acid bacteria, yeasts and molds, *Enterobacteriaceae*, and *Clostridium* spp.). On the other hand, it is unclear how these commercial frozen insect products are actually made, e.g., how long time has passed between killing and freezing these insects and under what environmental conditions. Nevertheless, after freezing, the insect samples' microbial loads are still can be considered moderate, which indicates that the freezing process cannot be relied on as to drastically reduce the number of microbes associated with the insects. Therefore, proper food processing practices are advisable to ensure the appropriate microbial qualities of the human dietary products made from such frozen insect raw materials.

### 3.2. Protein Content and Extraction

Total crude protein content determination by the Kjeldahl method (Table 2) revealed that field cricket had the highest proportion of protein (on dry weight basis) among the three frozen insects (about 52%) which is comparable to chicken meat's protein content (60% protein/dry weight [14]). However, the crude protein contents of bamboo caterpillar and silkworm were not far behind of the field cricket's (47.5 and 48.2%, respectively), underlying the potential value of these insects as novel nutritional protein sources.

Obtaining and separating the protein content from the insect body has its advantages and challenges from the food technologist's point of view, one of which is defatting the insects. Defatting procedures remove most of the lipids that form biological membranes therefore allowing easier access to both membrane and cytoplasmic proteins during a subsequent protein isolation/extraction process. In this work, two defatting methods were combined with protein isolation by isoelectric point precipitation at pH 3.5 or 4.5. The results presented in Table 3 show that in cases of cricket and bamboo caterpillar, the Soxhlet defatting method and the Folch lipid extraction combined with isoelectric point precipitation yielded similar protein content when compared at pH 3.5 or pH 4.5. More precisely, there was no significant difference in protein yield between the defatting methods and between isoelectric point precipitations at different pHs in field cricket and bamboo caterpillar samples. This indicates that from certain insect protein could be extracted in similar efficiency regardless of defatting method or isoelectric point precipitation pH, which might be a general rule but there can be exceptions such as in silkworm. As the results indicate, the protein yield in silkworm was higher at pH 3.5 in the Soxhlet defatted sample, as opposed to the Folch method treated sample where higher protein yield was obtained at pH 4.5. The reason behind this observation may lie in the lipid composition of the silkworm as well as its lipids association with more or less negatively charged proteins. For example, Soxhlet extraction with the less polar petroleum ether might expose a higher amount of more negatively charged proteins (isolated at pH 3.5) than the more polar solvents (dichloromethane and methanol) in Folch extraction. Similarly, the same polar solvents in Folch extraction might liberate a higher amount of less negatively charged proteins (precipitated at pH 4.5) than did petroleum ether in the Soxhlet method.

### 3.3. Subcritical Water Hydrolysis of Defatted Crickets

Isolated insect proteins, apart from being a novel option for human nutrition, may have other interesting features. To explore additional nutritional benefits of insect proteins, field crickets were subjected to another set of experiments based on the fact that they were found to have the highest relative protein content among the three examined frozen insect samples. To investigate other potential nutritional benefits, defatted field crickets were subjected to subcritical water hydrolysis (121 or 135°C for 15 or 30 minutes). The effect of these hydrothermal treatments on the total crude protein content is shown in Table 4. These results show that the total crude protein contents after hydrothermal treatments were lower than that of the untreated control (homogenized defatted cricket). This result can be attributed to the hydrothermal treatments which must have been produced a certain amount of cricket protein-derived oligopeptides and free amino acids [15], the latter being unreactive in the Lowry assay; hence, the decreased amount of peptide bonds resulted in less detected protein amounts.

### 3.4. Antioxidant Activities of Subcritical Water Hydrolysates of Field Cricket

DPPH radical scavenging activity assay and ferric reducing antioxidant power (FRAP) assay were carried out to test the potential antioxidant properties of hydrothermal cricket hydrolysates. The results of DPPH scavenging activities of defatted cricket hydrolysates shown in Table 4 indicated higher DPPH scavenging abilities of the hydrolysates than that of the control (untreated) defatted field cricket sample. The highest DPPH scavenging activity was detected in the hydrolysate treated at 135°C for 15 minutes followed by products of treatments at 121°C for 30 minutes, 121°C for 15 minutes, and 135°C for 30 minutes, respectively. The DPPH scavenging activity seemed to increase with higher temperature/longer time treatments except for 135°C 30 minutes treatment which resulted in the smallest activity among the treatments, although still higher than that of the control. Given the finding that defatted crickets contain relatively high amount of proteins, it seems likely that the effects of the applied hydrothermal treatments on the crickets' protein content at least partly responsible for the DPPH scavenging activity increase in the treated samples. It is known from other works [15–17] that even mild hydrothermal treatments can cause partial hydrolysis of proteins which results in the liberation of free amino acids and oligopeptides. It is also known that some free amino acids such as histidine [18] and tryptophan [19] as well as certain oligopeptides derived from hydrolysis of proteins exhibit free radical scavenging activity [20–22]. Therefore, taken the above observations together, we postulate that the applied hydrothermal treatments on defatted crickets released free amino acids and oligopeptides which contributed substantially to the DPPH scavenging activity of the samples that underwent hydrothermal treatment. However, it is also documented that the total polyphenol index in the water soluble extract of defatted crickets (*Acheta domesticus*) is relatively high among edible insects [2]. This finding implies that polyphenolic compounds may also contributed to the DPPH scavenging activities detected in those defatted crickets which are relatives of the field crickets (*Gryllus bimaculatus*). Additionally, it may also be possible that the applied hydrothermal treatments have positively influenced the bioavailability of polyphenolic compounds in our field cricket samples (similarly to that in defatted grape seeds [23] or in defatted orange peels [24]), which in turn were also involved in the observed increase of antioxidant activities of said cricket samples. In the light of the above observations, it can be speculated that on one hand increased hydrothermal treatment temperature and time duration increased antioxidant peptides and amino acid content in the treated samples as well as increased free polyphenol content as well. However, the observed low DPPH scavenging activity in the sample after the highest temperature-longest time hydrothermal treatment does not fit this pattern for uncertain reasons, for example something might interfere with the released antioxidant compounds. One possibility is that more intense subcritical water extraction might liberate other compounds as well (e.g., mono or oligosaccharides from chitin) that might enter chemical reactions with antioxidant compounds (e.g., Maillard reactions with amino acids) thus reducing the overall DPPH scavenging ability of the sample.

The results of the ferric reducing antioxidant power assay shown in Table 4 have similarities to DPPH radical scavenging activity results such as all the treated samples exhibited higher FRAP values than that of the untreated control. At both treatment temperatures, FRAP values were lower in the samples that underwent shorter treatment time (15 minutes), than in samples treated for a longer time (30 minutes). However, both samples treated at 121°C had higher FRAP values than that of treated at 135°C for 15 min, while the highest FRAP value was measured in the sample treated at 135°C for 30 minutes. Similar to that of DPPH radical scavenging activity discussed above, higher FRAP values in samples after hydrothermal treatments might be due to the cumulative effect of different compounds such as amino acids, oligopeptides, and polyphenols liberated from the defatted field cricket tissues by the treatments. It is likely that the hydrolytic effect of the hydrothermal treatments freed up amino acids such as cysteine and tryptophan with high FRAP values [25] as well as oligopeptides and polyphenolic compounds at various degrees and efficiencies, and these could effectively donate electrons or hydrogen to reduce the Fe^3+^-TPTZ complex. Our results also complement the finding of Di Mattia et al. [2] who reported relatively high FRAP value of defatted crickets (*Acheta domesticus*) among the tested edible insects.

## 4. Conclusions

This study focused on the microbial quality, defatting and protein extraction yield, and antioxidant properties of three edible insects whose general perception as dietary raw materials in western societies are mainly repulsive despite their relatively high protein content. It was found that a Thai commercial frozen product of either silkworm (*Bombyx mori*), bamboo caterpillar (*Omphisa fuscidentalis*), or field cricket (*Gryllus bimaculatus*) contained moderate microbial loads which potentially be further reduced by food preparation practices. Protein yields from the above insects after isoelectric point precipitation were compared and related to the defatting methods used. This revealed that the defatting method influenced the protein yield from only one of the three insects. Additionally, free radical scavenging and ferric reducing antioxidant power measurements were performed on subcritical water hydrolysates of defatted field crickets. The results of these measurements showed that the applied hydrothermal treatments were able to liberate compounds with antioxidant activities from the defatted insect samples.

## Figures and Tables

**Table 1:** Microbial analysis of frozen insects.

Frozen insects	Total aerobic mesophiles	Lactic acid bacteria	Yeasts and molds	*Enterobacteriaceae*	*Clostridium* spp.
Bamboo caterpillar	8.37 ± 0.05	5.21 ± 0.2	6.27 ± 0.23	6.56 ± 0.17	3.60 ± 0.2
Field cricket	7.94 ± 0.04	ND	5.59 ± 0.43	6.18 ± 0.11	3.19 ± 0.6
Silkworm	8.13 ± 0.17	ND	5.85 ± 0.22	4.71 ± 0.41	3.33 ± 0.19

Data (log cfu/g) are the mean ± standard deviations of three replicates. N.D.: not detected.

**Table 2:** Dry matter and protein content in selected insects.

Insect samples	Dry matter (%)	Protein (% of dry weight)
Bamboo caterpillar	32.69 ± 0.68	47.52 ± 0.55*b*
Field cricket	28.74 ± 1.04	52.08 ± 0.13*a*
Silkworm	23.49 ± 0.48	48.2 ± 0.84*b*

Mean values ± standard deviation (*n* = 3). Values with different letters within a column are significantly different (*p* < 0.05).

**Table 3:** Protein content (mg/mg defatted insect) of defatted insects after lipid removal at different pHs followed by isoelectric point precipitation.

Lipid removal	Soxhlet method		Folch method	
Isoelectric point precipitation	pH 3.5	pH 4.5	pH 3.5	pH 4.5
Bamboo caterpillar	0.153 ± 0.01	0.172 ± 0.002	0.167 ± 0.007	0.18 ± 0.008
Field cricket	0.676 ± 0.012	0.681 ± 0.024	0.734 ± 0.019	0.695 ± 0.004
Silkworm	0.462 ± 0.005^∗^	0.323 ± 0.017^∗^	0.265 ± 0.008^∗^	0.435 ± 0.019^∗^

Mean values ± standard deviation (*n* = 3). ^∗^Significantly different (*p* ≤ 0.05) from that isoelectric point precipitation conducted at different pH after the same lipid removal method.

**Table 4:** Protein content, DPPH radical scavenging activity, and FRAP of defatted field cricket before and after subcritical water hydrolyses.

Hydrothermal treatment of defatted field cricket	Protein (g/g of freeze-dried sample)	DPPH radical scavenging activity (%)	FRAP (mM Fe^2+^ eq./g)
Control (untreated)	1.23 ± 0.13	0.07 ± 0.08	0.30 ± 0.10
121°C, 15 min	0.86 ± 0.1^∗^	39.45 ± 1.72^∗^	0.97 ± 0.06^∗^
121°C, 30 min	0.66 ± 0.08^∗#^	44.68 ± 0.88^∗§^	1.01 ± 0.03^∗^
135°C, 15 min	0.63 ± 0.11^∗#^	45.51 ± 2.64^∗§^	0.79 ± 0.07^∗^
135°C, 30 min	0.61 ± 0.16^∗#^	28.41 ± 8.11^∗^	1.13 ± 0.04^∗^

Mean values ± standard deviation (*n* = 3). ^∗^Significantly different (*p* ≤ 0.05) from that of the Control. ^#^Significantly different (*p* ≤ 0.05) from that of the sample treated at 121°C for 15 minutes. ^§^Significantly different (*p* ≤ 0.05) from that of the sample treated at 121°C for 15 minutes and at 135°C for 30 min.

## Data Availability

The data used and/or analyzed in the study are available from the corresponding author on reasonable request.
